# Coagulation status of immune‐mediated polyarthritis in dogs

**DOI:** 10.1111/jsap.13838

**Published:** 2025-02-25

**Authors:** L. A. F. Packham, V. Black

**Affiliations:** ^1^ Langford Veterinary Services Small Animal Referral Hospital Langford UK; ^2^ Bristol Veterinary School University of Bristol Langford UK

## Abstract

**Objective:**

To describe the coagulation status of dogs with immune‐mediated polyarthritis.

**Materials and Methods:**

Hospital records at a single referral centre were retrospectively reviewed for dogs diagnosed with immune‐mediated polyarthritis. Cases were classified as hypercoagulable, normocoagulable, hypocoagulable, or hypercoagulable and hypocoagulable according to the results of viscoelastic testing performed at the time of diagnosis. Other data including signalment, body weight, age at diagnosis, haematological and biochemical laboratory findings, number of joints sampled and synovial fluid analysis, and short‐term outcome were recorded. Breed predisposition was defined through comparison to the hospital population and odds ratio calculation.

**Results:**

Thirty‐eight dogs were included with a median age of diagnosis of 4 years (range 9 months to 10 years). One dog (2.6%) was classified as hypercoagulable, 32 (84.2%) were classified as normocoagulable, four (10.5%) were classified as hypocoagulable and one dog (2.6%) had changes associated with both hypercoagulability and hypocoagulability.

**Clinical Significance:**

In contrast to other systemic inflammatory disorders, immune‐mediated polyarthritis was not commonly associated with either hypo‐ or hypercoagulability in this cohort of dogs.

## INTRODUCTION

Immune‐mediated polyarthritis (IMPA) may present with specific findings such as joint effusion, stiff gait, lameness and reluctance to rise. Or, non‐specific findings such as pyrexia, lethargy and inappetence (Johnson & Mackin, 2012). Though a proportion of cases have been shown to not present with either joint effusion or pyrexia (Rondeau et al., 2005). Erosive and non‐erosive polyarthropathies can be separated by means of radiographic imaging findings; whereby the presence of subchondral bone lysis is supportive of an erosive IMPA (*e.g*., rheumatoid arthritis and periosteal proliferative arthritis). The non‐erosive form can be further categorised into primary/idiopathic (type I), secondary/reactive (type II, III and IV), systemic lupus erythaematosus, drug or vaccine‐induced and breed‐associated. In the United Kingdom, non‐erosive idiopathic IMPA is the most common of the described forms (Perez et al., 2024). The disease may have a higher prevalence among certain breeds. Recent studies have suggested the Whippet, cocker spaniel, Miniature schnauzer, Cairn terrier, Hungarian Vizsla and Crossbreeds may be over‐represented for IMPA (Perez et al., 2024; Ravicini et al., 2023). The German shepherd dog had previously been reported as over‐represented (Bennett, 1987). However, this finding was not maintained in those more recent studies.

Pathophysiologically, non‐erosive IMPA results in the activation of the complement cascade, accumulation of antibody–antigen complexes within vessels of the synovium (Bennett, 1987; Tan et al., 2017) and subsequent recruitment of neutrophils to the joint space; resulting in a sterile inflammatory process. As well as local changes within the synovium, pro‐inflammatory cytokines such as interleukin‐6 (IL‐6) and acute‐phase proteins such as C‐reactive protein (CRP) have been demonstrated to be increased in the serum of canine patients with non‐erosive, idiopathic, IMPA (Bayer et al., 2023; Foster et al., 2014); suggesting a systemic inflammatory process. IL‐6 has also been identified to play an important role in the inflammatory pathway of rheumatoid arthritis in humans and has led to the development of targeted anti‐IL‐6 therapy (Akioka, 2019). Subcategories of IMPA types II to IV help to define potential associative causes of the antigenic stimulation subsequently leading to an arthropathy; though the cascade of events within the synovium, leading to a clinical and cytological diagnosis, remain the same. Type I has no identifiable associative disease and type II, III and IV define cases where there are putative associative diseases with separate pathology distant to the joint space.

There is a body of evidence to support the theory that many other systemic inflammatory disease processes can cause abnormalities to the coagulation status of canine patients. For example, immune‐mediated haemolytic anaemia (Goggs et al., 2012), protein‐losing nephropathy (Lennon et al., 2013), chronic enteropathy (Dixon et al., 2021), acute pancreatitis (Nielsen et al., 2019) and neoplasia (Andreasen et al., 2012) have all been demonstrated to cause hypercoagulability. Liver disease (Kelley et al., 2015) and disseminated intravascular coagulation (Wiinberg et al., 2008) have been demonstrated to cause hypocoagulability. Literature surrounding the coagulation status of canine patients with IMPA is lacking. The databases Web of Science, Ovid and Scopus have been searched with the following key words (dog* OR cani*) AND (IMPA OR polyarthritis OR IMPA) AND (coagulation OR coagulability) on June 10, 2024. This yielded three research articles in total: one of these is a single case report of a dog with *Bartonella henselae*; one describes 40 dogs with diseases reported to predispose to hypercoagulability (three of which had IMPA but were not individually commented on) and compares their coagulation status to 20 control dogs; and one measured thrombin‐antithrombin complex in both healthy dogs and dogs with various diseases (three of which had IMPA but were not individually commented on). No previous literature, based on this search, has specifically reported on the coagulation status of canine IMPA.

Viscoelastic testing assesses an individual patient's coagulation profile, in real time. Incorporating assessment of the ability for the blood to clot, the strength of the clot and the breakdown of the clot. Dependent on the equipment used, some methods of viscoelastic testing require addition of an activator (*e.g*., tissue plasminogen activator and kaolin) and others do not. Within veterinary medicine, viscoelastic testing is becoming well‐established as an additional tool in the assessment of global haemostasis in cats and dogs (McMichael & Smith, 2011). Whereby viscoelastic testing may have previously been limited to centres able to afford this laboratory equipment, and the requirement of personnel to run it, the development of more recent bedside monitors, using untreated whole blood, may allow wider utilisation of this test among the profession (Burton & Jandrey, 2020).

Thromboembolic events occur in veterinary medicine secondary to a variety of disorders (Ruehl et al., 2020). Although a definitive cause for a thromboembolic event is not always identified, the potential catastrophic sequalae from thromboembolic disease (secondary to coagulation disorders) justifies the need to further our knowledge in this area. To that end, the objective of this study is to define the coagulation status of non‐erosive IMPA in dogs. We hypothesise that, like other systemic inflammatory diseases, IMPA would result in a hypercoagulable state. A secondary objective was to identify breed predisposition through comparison to the hospital population and calculation of breed‐specific odds ratio.

## MATERIALS AND METHODS

### Inclusion criteria

Medical records of dogs referred to a university referral teaching hospital in the United Kingdom between January 1, 2021 and January 1, 2023 were reviewed. Medical records were searched (single operator) using the hospital's practice management system (RxWorks; Covetrus) for a diagnosis of “immune‐mediated polyarthritis”. Ethical approval was granted by the University's Animal Welfare and Ethics Review Body (VIN/21/015). Cases were included if a first‐time diagnosis of IMPA was made. This was defined as: joint fluid analysis from two or more joints demonstrating sterile neutrophilic inflammation, with a nucleated cell count >2 cells per 50× objective lens. For the purposes of the study objectives, cases must have had viscoelastic testing performed to be included. All cases required serum biochemistry as well as diagnostic imaging of the thorax and abdomen (either computed tomography of both regions, or thoracic radiographs and abdominal ultrasonography). Cases were then excluded if: erosive disease was identified on imaging (either computed tomography or radiography of a distal limb); they had received any immunosuppressive or antithrombotic therapy within 7 days prior to the diagnosis; and if they had not had a complete blood count performed (Siemens Advia 2120 as well as blood film analysis by a haematology laboratory scientist, as a requirement to assessment platelet numbers). Patients who were thrombocytopenic (<66,000 platelets ×10^9^/L, confirmed by blood film analysis and without platelet clumping) or had a haematocrit <30% or ≥60% (unless they were a breed known to have a physiological higher haematocrit; greyhounds, whippets (Uhríková et al., 2013) and dachshunds (Torres et al., 2014)) were excluded from the study as they have been demonstrated to significantly affect coagulation status (Smith et al., 2012).

### Data collection

Data gathered included: signalment, weight, date of diagnosis, age at diagnosis, travel history, vaccination status, clinicopathological testing results (including complete blood count, blood film analysis, serum biochemistry, serum cobalamin and CRP), diagnostic imaging findings, number of joints sampled, arthrocentesis cytology, viscoelastic testing results, duration of clinical signs, medications administered within 1 week of presentation, survival to discharge and survival at 3 months.

### Classification

Cases were defined into subcategories of IMPA: type I, II, III or IV. Type I cases lacked any other identifiable disease. Type II cases had an infectious process identified at any site other than within the synovium. Infectious disease testing was performed where this was considered clinically appropriate and was not included within the inclusion criteria. All travelled cases had infectious disease testing performed (this included individual or combinations of: Idexx SNAP 4Dx^™^ (all cases), *Leishmania* antibody ELISA and *Brucella canis* serology). Type III cases had a concurrent enteropathy, defined by analysis of a combination of their clinical history, ultrasonographic findings of the gastrointestinal tract and their serum cobalamin value. Measurement of serum cobalamin was performed in cases where this was considered clinically appropriate and was not included within the inclusion criteria. Type IV cases had a neoplastic process identified at the time of IMPA diagnosis.

### Coagulation status

**FIG 1:**
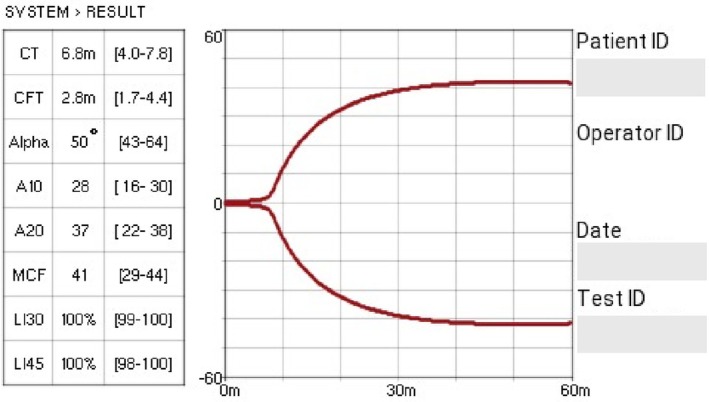
An example of an Entegrion VCM Vet^™^ trace (here, the values “m” refer to minutes; converted to seconds for all cases within the current study).

Coagulation status was assessed via a point‐of‐care viscoelastic monitoring system; Entegrion VCM Vet^™^. Daily quality assurance checks performed on the device, both internal (self‐check internal quality control) and external (daily use of System Check Cartridges, to analyse mechanical function and calibration ratios) were performed throughout the study period (as is usual practice within the hospital) and as per the operator's manual, servicing of the device is not required within 5 years. As described within the operator's manual, the Entegrion VCM Vet^™^ contains two glass discs mounted on parallel flexible plastic arms, forming a capillary to hold the test sample. The glass discs trigger clotting of the blood sample via contact activation. A motor drives *one* of these flexible arms, causing it to oscillate back and forth. An optical sensor analyses the movement of both arms. As the blood progresses through clotting, retraction and lysis, the device analyses the movement of the un‐driven arm against that of the driven arm and calculates, in real time, parameters to characterise the clot and it is displayed as a graphical trace. An example of this trace is given in Fig. 1.

Data were collected of four parameters from this test: clot time, clot formation time, alpha angle and maximum clot formation. Clot time (CT) is measured from the start of the test to the time that an amplitude of 1% above baseline is reached. Clot formation time (CFT) is the time point when the amplitude of the clotting signal reaches 10%. The alpha angle describes the kinetics of the clot and the rate of fibrin formation. Maximum clot formation (MCF) is a measure of clot firmness and clot quality, measured at maximum amplitude before the onset of clot fibrinolysis. Reference intervals were as those defined in a previous study (Buriko et al., 2020), and were as follows: CT 241 to 470 seconds, CFT 104 to 266 seconds, alpha angle 43° to 64° and MCF 29 to 44 VCM Units. Hypercoagulability was defined as a 25% deviation from the established reference intervals, of two or more parameters, in the appropriate direction (decreased CT, increased alpha angle and increased MCF). Hypocoagulability was defined as a 25% deviation from the established reference intervals, of two or more parameters, in the appropriate direction (increased CT, decreased alpha angle and decreased MCF).

### Statistical analysis

Descriptive data were expressed as median values with total ranges, and categorical data were expressed as individual frequencies. Breed predisposition, within this study, was calculated utilising the total number of dogs seen by the referral hospital during the study period with and without IMPA, as well as the number of each individual breed with and without IMPA. The odds ratio calculation was performed using an online odds ratio calculator (https://www.medcalc.org/calc/odds_ratio.php) and the 95% confidence intervals and p‐values were included. As with previous studies employing this method (Allerton et al., 2018), breeds were considered to be significantly predisposed to IMPA if the lower 95% confidence interval did not cross below 1.0. Breeds that were only represented once for IMPA were not included in this breed predisposition analysis to reduce likelihood of a type 1 error.

## RESULTS

### Study population

During the period of data collection, a total of 60 dogs were identified via the practice management system database search; these 60 cases were assessed for eligibility to be included within the study. All 60 dogs were identified to have a first‐time diagnosis of IMPA and 42 of these had viscoelastic testing performed at the time of presentation. Of these 42 dogs, one case was identified to have erosive change on radiography and was excluded and two had received immunosuppressive or antithrombotic therapy within 7 days of the diagnosis and were excluded. One case was excluded due to a haematocrit of <30%. No cases were excluded due to platelet count.

Thirty‐eight cases of dogs with non‐erosive IMPA met the inclusion criteria. There were 22 female dogs (16 neutered and six entire) and 16 male dogs (six neutered and 10 entire). The median age at time of diagnosis was 4 years old, with a range from 9 months to 10 years old. The median weight at time of diagnosis was 15.3 kg, with a range from 4.5 to 72 kg. The most commonly represented breeds were Whippet (*n* = 9), cocker spaniel (*n* = 5), Crossbreed (*n* = 3), German shepherd dog (*n* = 3), English springer spaniel (*n* = 3) and shih‐tzu (*n* = 2). All other breeds were represented once. For all breeds presenting more than once, the associated odds ratio and 95% confidence intervals are shown in Table 1.

**Table 1:** All breeds presenting more than once with the associated odds ratio and 95% confidence intervals

Breed	Number of IMPA cases within this breed/total number of this breed presenting within study period	Odds ratio	Lower/upper 95% confidence interval	P value
Whippet	**9/118**	**23.9**	**11.0/51.2**	**P < 0.0001**
Cocker spaniel	**5/432**	**2.9**	**1.1/7.4**	**P = 0.0293**
German shepherd dog	**3/169**	**4.3**	**1.3/14.1**	**P = 0.0162**
English springer spaniel	3/305	2.3	0.7/7.6	P = 0.1628
Crossbreed	3/283	2.7	0.8/8.7	P = 0.1056
Shih‐tzu	**2/92**	**5.2**	**1.2/21.9**	**P = 0.0249**

Those in bold have a lower 95% confidence interval that did not cross below 1.0

Two of 38 dogs had travel history outside of the United Kingdom (Republic of Ireland and France). Thirty‐six of the 38 dogs had available vaccination history, 1/36 dogs received a vaccine within 30 days of diagnosis. One dog had received immunosuppressive medication 8 weeks prior to diagnosis, eight dogs had received immunosuppressive medication between 8 weeks and 8 years prior to diagnosis, and 29 dogs had never received any immunosuppressive medications at any time point.

### Clinical findings

The median temperature on presentation was 39.5°C, with a range of 38.1 to 40.8°C. The median duration of clinical signs, prior to presentation, was 1 month with a range of <1 month to 6 months. CRP was not available for one dog. The median CRP value was 128.2 mg/L (<5 to 365.3 mg/L). Six cases had a CRP within reference interval (0 to 10 mg/L). Of these six cases with a normal CRP, all six had received a non‐steroidal anti‐inflammatory medication at the time, or within 1 week, of presentation. In all cases, the minimum number of joints sampled was four, with a range of four to eight joints. The location of arthrocentesis in all cases was: carpus, elbow, tarsus and stifle.

### Classification

Of the 38 dogs that met the inclusion criteria, 28 cases were defined as type I IMPA; three cases were define as type II IMPA; six cases were define as type III IMPA; and one case was defined as type IV IMPA.

### Coagulation status

Of the 38 dogs that met the inclusion criteria one was classified as hypercoagulable, 32 were classified as normocoagulable, four were classified as hypocoagulable and one case had changes associated with both hypercoagulability and hypocoagulability. The distribution of IMPA classification according to coagulation status is displayed in Table 2. Full results of viscoelastic testing for all dogs is presented in the Table S1.

**Table 2:** Distribution of subcategory of IMPA and associated coagulation status

	Hypocoagulable	Normocoagulable	Hypercoagulable	Both hyper‐ and hypocoagulable
Type I	2	25	0	1
Type II	0	3	0	0
Type III	2	3	1	0
Type IV	0	1	0	0

### Outcome

All cases survived to discharge. 36/38 cases were alive 3‐month after diagnosis. Of the two cases that did not survive to 3 months post‐discharge, both were classified as normocoagulable at diagnosis. One case was a Miniature schnauzer that died suddenly, 48 hours after discharge and the other was a Whippet that was euthanased following suspected infection of an orthopaedic implant placed 3 years prior to IMPA diagnosis. The single hypercoagulable case had a concurrent diagnosis of chronic enteropathy (compatible clinical signs, intestinal changes identified on ultrasound and decreased serum cobalamin). Treated with immunosuppressive dose of corticosteroid (prednisolone), this case presented for a routine recheck 6 weeks following initial discharge and repeat viscoelastic testing was reported as normocoagulable. The single case that demonstrated both hypo‐ and hypercoagulable tendencies was alive at 3 months.

## DISCUSSION

The results of this study suggest that, in contrast to other systemic inflammatory disorders, canine patients with non‐erosive IMPA are rarely hypercoagulable. Furthermore, in this cohort of dogs, coagulability changes were uncommon and hypocoagulability was more common than hypercoagulability.

The cause of hypercoagulability in various other immune‐mediated disease has been purported to include complex relationship changes between pro‐coagulant and anticoagulant factors, endothelial injury, significant tissue factor expression and platelet activation (Swann et al., 2019). Disease in which systemic inflammation is present can cause a pro‐coagulant state (Esmon, 2004). However, despite IMPA commonly presenting with increased CRP (suggestive of a systemic inflammatory response), the results of this current study do not agree with this statement. Possible explanations for these findings are that the above physiological changes may not occur in IMPA; that their relationship differs significantly enough that a hypercoagulable state is not a likely sequalae; or, that the utilised technique is insufficient to appropriately detect hypercoagulability in this cohort of dogs. Hypercoagulability has been demonstrated in a population of dogs with an immune‐mediated disease in which it was suspected, using the same device as in this current study (Dionne et al., 2023). However, the use of positive controls used within the same local setting, on the same machine, would be an ideal way to demonstrate the ability to detect hypercoagulability appropriately.

Compared to disease such as immune‐mediated haemolytic anaemia and protein‐losing nephropathy, the intravascular pathology that contributes to a hypercoagulable state may differ with IMPA as, despite having similar characteristics of a systemic disease (*e.g*., increased CRP), it specifically manifests within the synovium. A disease most similar to canine non‐erosive IMPA in humans is juvenile idiopathic arthritis, specifically systemic‐onset juvenile idiopathic arthritis (sJIA). The pro‐inflammatory cytokine tumour necrosis factor alpha (TNFα) has been demonstrated to be increased in both blood and synovial fluid in human patients with sJIA (Kutukculer et al., 1998). Like sJIA, canine patients with IMPA have also been shown to have an increase of TNFα in the synovium (Hegemann et al., 2005). However despite this, as with our study, there is currently little evidence to suggest that hypercoagulability is a significant complication of human patients with sJIA (Cimaz, 2016). These findings may suggest a likeness of these two disease processes. Anti‐TNFα drugs signal a recent progression in biologic therapy in human medicine (Scardapane et al., 2012). Further studies are needed to evaluate the role of TNFα in canine patients with IMPA.

The single hypercoagulable case was a 9‐month‐old Labrador. Clinical signs had been present for 6 months prior to diagnosis and the CRP was 352.9 mg/L (the third highest of the study). This patient was classified as type III IMPA. As described above, enteropathies have been demonstrated to cause hypercoagulability (Dixon et al., 2021; Goodwin et al., 2011; Wennogle et al., 2021) and in this particular case may indeed be the cause of the patient's coagulation status rather than IMPA. The patient returned 6 weeks after diagnosis for a routine follow‐up; repeat viscoelastic testing was documented as normocoagulable, the patient was in cytological remission following repeat arthrocentesis and was alive at 3 months post‐discharge. Of the four cases that were hypocoagulable two were categorised at type I IMPA and two were categorised as type III IMPA. Hypocoagulability has been identified in otherwise apparently healthy canine patients (Dionne et al., 2023) suggesting that hypocoagulability as defined by viscoelastic testing alone may not infer clinical significance. Further supported by the current lack of consensus, by viscoelastic testing, on a definition of hypocoagulability (Hanel et al., 2014). All four cases of hypocoagulability, as defined by this study, were alive at 3 months post‐discharge.

As has been demonstrated in other studies (Rondeau et al., 2005) a small number of canine patients with IMPA will present without typical clinicopathological or examination findings (*e.g*., normal CRP values and normothermia, respectively). It was interesting to note that all six cases in this study presenting with a CRP within reference interval had received a non‐steroidal anti‐inflammatory medication within 7 days of the diagnosis. Whether the attenuation of the production of acute‐phase proteins (such as CRP) can occur during administration of non‐steroidal anti‐inflammatory drugs due to the inhibition of cyclooxygenase enzymes, and subsequent decreased production of pro‐inflammatory cytokines, may depend on the type of non‐steroidal anti‐inflammatory used (Tarp et al., 2012). However, current veterinary research suggests that non‐steroidal anti‐inflammatory drugs do not significantly affect serum CRP values (Bennett et al., 2013; Borer et al., 2003; Kum et al., 2013). All six of these cases with a normal CRP value were reported as normocoagulable, were type I IMPA and were alive at 3 months post‐discharge. There were five other cases that had also received non‐steroidal anti‐inflammatory medication and did have an increase in CRP (median 102.4 mg/L, range 12.2 to 142.7 mg/L). Non‐steroidal anti‐inflammatories may affect platelet function (Mullins et al., 2012) but studies have demonstrated that they exert no significant effect on the results of viscoelastic testing in dogs (Wang et al., 2022; Zanuzzo et al., 2015).

In this study, the Whippet was the most numerous breed and was identified to be over‐represented compared to the hospital population during the same data collection time period, with an odds ratio of 23.9 (95% confidence interval 11.0 to 51.2). The Whippet is a breed that has previously been demonstrated to be over‐represented for non‐infectious inflammatory disease (Black et al., 2019; Rose et al., 2014) and IMPA in particular (Ravicini et al., 2023). The frequency with which this breed is represented may suggest a genetic alteration in the immune response to certain common stimuli. However, a recent study of 84 cases of canine IMPA found no increase in predisposition of the Whippet (Perez et al., 2024). Cocker spaniels, German shepherd dog and the shih‐tzu were also shown to be over‐represented in the current study. cocker spaniels (Ravicini et al., 2023) and German shepherd dog (Bennett, 1987) have been previously described as over‐represented for IMPA. The shih‐tzu breed has not been described as over‐represented previously, but IMPA has been reported in this breed (Jung et al., 2006). If there were low numbers of shih‐tzus presenting to the hospital within the study period, this may reflect a type I error. Greyhounds have been shown to have unique viscoelastic parameters compared to non‐Greyhound breeds (Chang et al., 2021). Similar differences have also been demonstrated in Irish Wolfhounds (Davis et al., 2024); these findings may suggest the need to consider breed‐specific definitions of coagulability (particularly within sighthounds). There were no Greyhound or Irish Wolfhounds within the dataset of the present study.

There are a number of limitations within the current study. With particular regards to the VCM Vet^™^ and definition of coagulation status, these include inter‐operator variability, environmental conditions and no standardised time between venepuncture and testing. However, all staff using this machine are trained to use it appropriately and all samples were analysed within the suggested time frame in the operator's manual. There was no control population and only one viscoelastic test was performed per patient; this may have decreased precision and could be mitigated in future studies by performing serial viscoelastic testing. There are established reference intervals, for dogs, for the device used in this study (Buriko et al., 2020) and this device has been shown to have good agreement between units (Hennink et al., 2023). However, predetermined reference intervals may not be appropriate for every hospital population, due in part to the recommendation of local validation and subsequent studies may consider the development of individual hospital‐specific reference intervals.

Further limitations include the relatively small sample sized used in this study; cases were collected from the time point with which the current viscoelastic testing device was utilised by the hospital and therefore earlier cases would have met the exclusion criteria of this study. Furthermore, 38 cases over a 2‐year time period suggest that a prospective study should be multicentred to generate significantly more case numbers in an acceptable time frame. Fundamental to its retrospective nature, a further limitation to the current study is lack of standardisation across all cases with regards to patient variables. Particularly medication; including non‐steroidal anti‐inflammatories and corticosteroids. The effects of corticosteroids on coagulation status may last longer than 7 days, shown to last up to 6 weeks in one study (Rose et al., 2011) and therefore 7 days may be an inadequate washout period for prednisolone. Assessment and statistical analysis, of the length and severity of clinical signs and their relationship with coagulation status was not performed within this study. The development of a clinical scoring tool for assessment of disease severity in canine IMPA would require validation and is an area of suggested future research.

Further limitations of this study, in a broader sense, include the clinical application of the term “hypercoagulable” Clinically, patients who demonstrate hypercoagulable *tendencies* may differ from those described in a hypercoagulable *state*. A widely accepted consensus on the definition of the term “hypercoagulable”, specifically with regards to the use of viscoelastic assays is lacking; this is also true within human medicine (Brown et al., 2020). Previous veterinary studies have used a wide variety of individual definitions to report on hypercoagulability. This includes: statistical analysis to a control population (Goggs et al., 2012), a single coagulation parameter outside a predetermined cut‐off (Kelley et al., 2015), two or more coagulation parameters outside of the reference interval (Adamantos et al., 2015), a G value (a calculated parameter derived from maximum amplitude on thromboelastography) outside the reference interval (Mitsui et al., 2024) or a defined percentage outside the reference interval (Dixon et al., 2021). In the present study, a 25% deviation of two or more coagulation parameters was used to define hypercoagulability and hypocoagulability.

The risk of thromboembolism, a common sequalae of hypercoagulability in patients with immune‐mediated disease such as immune‐mediated haemolytic anaemia (Weinkle et al., 2005) and protein‐losing nephropathy (Lennon et al., 2013), is considered high enough in both these diseases to warrant antithrombotic drugs as part of standard therapy (IRIS Canine GN Study Group Standard Therapy Subgroup et al., 2013; Swann et al., 2019). It is therefore an important aspect of disease management, when necessary, to mitigate these risks where at all possible and to allow conversations with the pet owner to broach difficult subjects of morbidity and mortality. In conclusion of this study, it is considered unlikely that patients with non‐erosive IMPA will benefit from routine antithrombotic therapy and the risk of hypercoagulability is low. However, patients may still be at risk of sudden death even when demonstrating normocoagulability on viscoelastic testing (as one patient in this study showed), which highlights our need to further understand individual causes of coagulation variation. Prospective studies are needed with larger case numbers to identify if the findings in this study are upheld through statistical analysis, and to also identify those patients at higher risk of sudden death.

### Author contributions

**L. A. F. Packham:** Data curation (lead); formal analysis (lead); investigation (equal); methodology (equal); writing – original draft (lead); writing – review and editing (equal). **V. Black:** Conceptualization (lead); data curation (supporting); formal analysis (supporting); investigation (equal); methodology (equal); supervision (lead); writing – review and editing (equal).

### Conflict of interest

None of the authors of this article has a financial or personal relationship with other people or organisations that could inappropriately influence or bias the content of the paper.

## Supporting information


Table S1.

## Data Availability

The data that support the findings of this study are available from the corresponding author upon reasonable request.
