# Supplementary material for: Coagulation status of immune‐mediated polyarthritis in dogs
Source: J Small Anim Pract. 2025 Feb 25;66(7):443–50. doi: 10.1111/jsap.13838 (PMC12232344; doi:10.1111/jsap.13838)
Supplement: Supplementary file 1 — Table S1. [file JSAP-66-443-s001.docx]

| **Case** | **Signalment** | **Breed** | **Weight at diagnosis (kg)** | **Age at diagnosis** | **Length of clinical signs pre-diagnosis (months)** | **Temperature at presentation (°C)** | **Travel history** | **IMPA Type** | **Hct (%)** | **PLT (x10^9/L)** | **CRP (mg/L)** | **CT (241 - 470 seconds)** | **CFT (104 - 266 seconds)** | **Alpha angle (degrees)** | **MCF (VCM Units)** | **Coagulability** | **Survived to discharge** | **Alive 3 months after discharge** |
| --- | --- | --- | --- | --- | --- | --- | --- | --- | --- | --- | --- | --- | --- | --- | --- | --- | --- | --- |
| 1 | FN | Whippet | 11.2 | 3y 1m 21d | 3 | 39.2 | No | 1 | 54.6 | 209 | 21.8 | 482 | 269 | 47 | 30 | Normal | Yes | Yes |
| 2 | FN | SBT | 21.9 | 5y 1m 9d | <1 | 40.5 | No | 1 | 46.8 | 275 | 154.4 | 598 | 184 | 50 | 35 | Normal | Yes | Yes |
| 3 | ME | Crossbreed | 28.4 | 6y 5m 1d | <1 | 39.4 | No | 1 | 48.2 | 54 (clumping)† | 150.2 | 458 | 262 | 45 | 31 | Normal | Yes | Yes |
| 4 | MN | Boxer | 31.4 | 1y 4m 24d | 2 | 38.5 | No | 1 | 51.2 | 255 | <5 | 499 | 155 | 59 | 39 | Normal | Yes | Yes |
| 5 | FN | Crossbreed | 16.4 | 4y 8m 1d | <1 | 39.6 | No | 1 | 36.3 | 108 | 308.6 | 356 | 192 | 50 | 32 | Normal | Yes | Yes |
| 6 | FE | Shih Tzu | 4.7 | 11m 3d | 2 | 40 | No | 1 | 35.4 | 86 | 361.9 | 664 | 250 | 38 | 37 | Normal | Yes | Yes |
| 7 | FE | Miniature Schnauzer | 10 | 10y 1m 3d | <1 | 40.2 | No | 1 | 45.8 | 140 |  | 628 | 279 | 43 | 27 | Normal | Yes | No |
| 8 | ME | Flat Coat Retriever | 41 | 4y 3m 1d | <1 | 38.7 | No | 1 | 42.4 | 301 | 145.1 | 429 | 122 | 60 | 46 | Normal | Yes | Yes |
| 9 | MN | Whippet | 11.3 | 8y 6m 25d | <1 | 40.4 | No | 1 | 47 | 90 | 155.2 | 428 | 207 | 55 | 31 | Normal | Yes | Yes |
| 10 | MN | Whippet | 16.9 | 1y 11m 1d | <1 | 39.2 | No | 1 | 59.8 | 46 (clumping)† | 31.9 | 461 | 263 | 46 | 30 | Normal | Yes | Yes |
| 11 | FN | GSD | 29.6 | 5y 10m 30d | 6 | 40.3 | No | 1 | 39.2 | 235 | 63.8 | 486 | 153 | 59 | 39 | Normal | Yes | Yes |
| 12 | FN | Whippet | 12.6 | 9y 11m 9d | 3 | 39.5 | No | 3 | 56.5 | 125 | 115.9 | 635 | 384 | 27 | 29 | Hypo | Yes | Yes |
| 13 | FN | GSD | 25.8 | 5y 0m 9d | 1 | 39.8 | No | 1 | 44.5 | 319 | 104.9 | 254 | 161 | 53 | 47 | Normal | Yes | Yes |
| 14 | FN | Cocker Spaniel | 12.3 | 6y 0m 10d | 3 | 39.5 | No | 1 | 39.7 | 370 | 16.4 | 629 | 100 | 68 | 41 | Normal | Yes | Yes |
| 15 | ME | Labrador | 14.3 | 9m 1d | 6 | 40.2 | No | 3 | 41.3 | 230 | 352.9 | 432 | 76 | 71 | 67 | Hyper | Yes | Yes |
| 16 | FE | Cocker Spaniel | 11.2 | 2y 5m 10d | 6 | 39.4 | No | 1 | 51.5 | 341 | <5 | 7 | 2.6 | 55 | 39 | Normal | Yes | Yes |
| 17 | FE | ESpSp | 14.4 | 4y 3m 20d | 3 | 38.1 | No | 1 | 39.3 | 412 | 108.1 | 7.4 | 1.8 | 64 | 43 | Normal | Yes | Yes |
| 18 | MN | Whippet | 12.1 | 3y 8m 12d | 2 | 39.5 | No | 1 | 43.8 | 164 | 112.2 | 8.8 | 3.1 | 52 | 37 | Normal | Yes | No |
| 19 | FN | Bouvier Des Flanders | 41.6 | 8y 10m 2d | <1 | 40.7 | Yes | 3 | 38.7 | 252 | 365.3 | 6.8 | 2.8 | 50 | 41 | Normal | Yes | Yes |
| 20 | FN | Pomeranian | 12.9 | 1y 0m 8d | 1 | 40.3 | No | 1 | 45.2 | 233 | 176.7 | 2.2 | 5.8 | 39 | 23 | Mixed | Yes | Yes |
| 21 | FN | Whippet | 12.2 | 2y 0m 18d | 1 | 38.7 | No | 1 | 62.2 | 131 | <5 | 6.5 | 5.5 | 42 | 26 | Normal | Yes | Yes |
| 22 | ME | Akita | 42.9 | 1y 0m 28d | <1 | 39.1 | No | 3 | 42.5 | 140 | 134.6 | 3.2 | 3.1 | 48 | 43 | Normal | Yes | Yes |
| 23 | FE | GSD | 22.7 | 11m 26d | <1 | 38.9 | Yes | 2 | 43.1 | 384 | 166.1 | 9.9 | 2.8 | 52 | 42 | Normal | Yes | Yes |
| 24 | FN | Cocker Spaniel | 10.4 | 2y 0m 13d | 2 | 40.8 | No | 1 | 35.5 | 471 | 191.6 | 4 | 3 | 44 | 56 | Normal | Yes | Yes |
| 25 | ME | Whippet | 16.25 | 2y 6m 8d | 3 | 39.1 | No | 1 | 57.9 | 145 | <5 | 8.2 | 4.6 | 44 | 31 | Normal | Yes | Yes |
| 26 | ME | Siberian Husky | 22.1 | 10m 3d | <1 | 39.8 | No | 1 | 35.4 | 148 | 183 | 14.6 | 6.7 | 25 | 32 | Hypo | Yes | Yes |
| 27 | FN | Cocker Spaniel | 13.7 | 3y 6m 17d | <1 | 40.3 | No | 1 | 37.1 | 115 | 87.87 | 10.4 | 5 | 39 | 19 | Hypo | Yes | Yes |
| 28 | ME | ESpSp | 16.4 | 1y 0m 1d | 3 | 39.8 | No | 3 | 43.1 | 221 | 97.54 | 11.9 | 5.5 | 32 | 31 | Hypo | Yes | Yes |
| 29 | ME | British Bulldog | 21.7 | 9m 3d | 1 | 39.7 | No | 2 | 37.7 | 146 | 148.28 | 7.1 | 3.6 | 43 | 36 | Normal | Yes | Yes |
| 30 | FN | Whippet | 11.6 | 7y 2m 20d | 6 | 39.1 | Yes | 1 | 46.6 | 374 | 69.01 | 9.2 | 3.1 | 54 | 35 | Normal | Yes | Yes |
| 31 | MN | Border Collie | 21.4 | 5y 1m 2d | <1 | 38.3 | No | 3 | 41.3 | 161 | 24.84 | 8.5 | 2.6 | 56 | 39 | Normal | Yes | Yes |
| 32 | ME | Cocker Spaniel | 10.7 | 6y 0m 14d | 4 | 38.7 | No | 1 | 45.9 | 343 | <5 | 6.3 | 2.5 | 59 | 40 | Normal | Yes | Yes |
| 33 | FN | Miniature Dachshund | 4.5 | 2y 0m 2d | 1 | 38.9 | No | 1 | 55.7 | 417 | 102.4 | 3.4 | 4.3 | 38 | 41 | Normal | Yes | Yes |
| 34 | FE | Mastiff | 72 | 1y 5m 21d | <1 | 39.5 | No | 1 | 42.5 | 466 | 142.7 | 8 | 3 | 52 | 37 | Normal | Yes | Yes |
| 35 | ME | Whippet | 18.1 | 6y 3m 3d | 6 | 39 | No | 1 | 46.3 | 495 | <5 | 6.6 | 2 | 63 | 46 | Normal | Yes | Yes |
| 36 | FN | Shih Tzu | 4.8 | 9y 8m 13d | <1 | 39.2 | No | 1 | 39.7 | 356 | 128.2 | 7 | 1.8 | 62 | 55 | Normal | Yes | Yes |
| 37 | FN | Crossbreed | 10.6 | 6y 10m 3d | <1 | 39.3 | No | 4 | 32.2 | 183 | 155 | 6.2 | 1.9 | 62 | 46 | Normal | Yes | Yes |
| 38 | MN | ESpSp | 23.3 | 5y 1m 5d | 3 | 38.4 | No | 2 | 48.4 | 252 | 12.2 | 2.7 | 2.9 | 53 | 39 | Normal | Yes | Yes |

Supplementary table 1: SBT – Staffordshire Bull Terrier; GSD – German Shepherd Dog; ESpSp – English Springer Spaniel; IMPA – immune-mediated polyarthritis; Hct – haematocrit; PLT – Platelet count; CRP – C-reactive protein; CT – Clot time; CFT – Clot formation time; MCF – Maximum clot formation. † blood film analysis confirms presence of platelet clumping.
